# Ex vivo conditioning with IL-12 decreases T cell sensitivity to intratumoral INF-γ-induced apoptosis following adoptive transfer

**DOI:** 10.1186/2051-1426-3-S2-P12

**Published:** 2015-11-04

**Authors:** C Marcela Diaz-Montero, Charles Tannenbaum, Patricia Rayman, Paul Pavicic, Jin Sub Kim, Marc Ernstoff, James Finke

**Affiliations:** 1 Cleveland Clinic Foundation, Cleveland, OH, USA

## Background

In order to induce significant tumor regression T cells must effectively recognize and kill target cells. Secretion of IFN-γ is considered a key effector function of activated CD8^+^ T cells via induction of apoptosis. Thus programming T cells to secrete high levels of IFN-γ after adoptive transfer could represent a therapeutically effective anti-cancer intervention.

## Methods

We previously demonstrated that naïve CD8^+^ T cells exposed to IL-12 during antigenic priming (Pmel^Ag+12^) provided superior anti-tumor activity after transfer when compared to cells activated in the presence of antigen alone (Pmel^Ag^). In this setting, tumor regression was associated with sustained levels of intra-tumoral IFN-γ. Expression analysis using total tumor RNA showed elevated expression of IFN-γ responsive genes such as IP-10, MCP-1, MIG, and MIP-1α. Even without IL-12 stimulation during *ex vivo* antigenic priming, Pmel cells were able to initially reach the tumor and secrete high levels of IFN-γ. However, by day 7 after adoptive transfer tumors in mice that received Pmel^Ag^ were significantly larger than those in mice injected with Pmel^Ag+12^. Failure to maintain intra-tumoral levels of IFN-γ was associated with a decrease in the frequency of tumor infiltrating Pmel^Ag^. We hypothesized that high levels of IFN-γ had a detrimental effect on Pmel^Ag^, via induction of apoptosis. IFN-γ is a multifunctional cytokine that induces a variety of contrasting cell responses such as proliferation or cell death. The cellular response to an IFN-γ stimulus depends on the specific receptor being activated, with IFN-γR1 inducing proliferation and IFN-γR2 inducing apoptosis.

## Results

We tested the hypothesis that the ability of T cells to survive *in vivo* after adoptive transfer was dependent on their susceptibility to IFN-γ-induced apoptosis. Real time PCR revealed that the expression levels of IFN-γR1 and IFN-γR2 immediately following antigen or antigen+ IL-12 priming were similar, though by 4d post adoptive transfer the tumor-infiltrating Pmel cells stimulated with antigen alone had 10 fold higher levels of IFN-γR2 than tumor associated Pmel^Ag+IL-12^.

## Conclusions

These results suggest that the enhanced anti-tumor activity of Pmel^Ag+IL-12^ might be due to their decreased sensitivity to IFN-γ-induced apoptosis. Thus inhibiting IFN-γ-induced activation induced cell death (AICD) by down-regulating IFN-γR2 expression on T cells may represent a novel mechanism by which IL-12 enhances anti-tumor activity.

